# Polysomnographic Sleep Parameters: Novel Digital Biomarkers for Developing Dementia

**DOI:** 10.1093/geroni/igaa057.534

**Published:** 2020-12-16

**Authors:** Javad Razjouyan, Sara Nowakowski, Amir Sharafkhaneh, Mark Kunik, Aanand Naik

**Affiliations:** 1 Center for Innovations in Quality, Effectiveness, and Safety (iQuest), Houston, Texas, United States; 2 Baylor College of Medicine, Houston, Texas, United States; 3 Michael E. DeBakey VA Medical Center, houston, Texas, United States

## Abstract

Neuroprotection, early diagnosis, and behavioral intervention are priorities for dementia research. Sleep is emerging as an important potential remediable risk factor. Polysomnography using various digital parameters, qualitatively and quantitively, evaluates sleep. In this study, we examined whether sleep parameters derived from attended overnight polysomnography (PSG) studies are associated with developing dementia. We retrieved 61,165 free-text PSG reports from the VA national electronic health records from 2000 to 2019. Patients with dementia diagnosis up to one-year after PSG were excluded. Patients who developed dementia >1 year after PSG (Dem) were classified using all-cause dementia ICD-9/10 codes documented on two separate visits starting a year after the PSG until the end of 2019 in a 1-year sliding period. Patients with no ICD-9/10 dementia codes (NDem) were propensity matched 1:1 for age, gender, race, ethnicity, BMI, and Charlston comorbidity index to the Dem group (n=1,534). We used natural language processing to identify sleep onset latency (SOL), total sleep time (TST), and apnea-hypopnea index (AHI). Univariate analysis was used to compare the groups. TST (254 v 266m, p=0.001) were significantly shorter and SOL (28 v 31m, p=0.047) were significantly prolonged in Dem compared to NDem. The odds ratio of individuals with an AHI ≥ 15 was significantly higher in Dem compared to NDem group (1.18, 95%CI: 1.04-1.35). Patients with incipient dementia exhibited longer SOL, shorter TST, and a greater proportion had moderate-to-severe apnea compared to patients without dementia. These objective sleep parameters may serve as potential biomarkers for patients at risk for developing dementia.

